# Young age increases risk for lymph node positivity in gastric cancer: A Chinese multi-institutional database and US SEER database study

**DOI:** 10.7150/jca.37531

**Published:** 2020-01-01

**Authors:** Yuming Jiang, Weicai Huang, Jingjing Xie, Zhen Han, Chuanli Chen, Sujuan Xi, Zepang Sun, Yanfeng Hu, Liying Zhao, Jiang Yu, Tuanjie Li, Zhiwei Zhou, Shirong Cai, Guoxin Li

**Affiliations:** 1 Department of General Surgery, Nanfang Hospital, Southern Medical University, 1838 North Guangzhou Avenue, Guangzhou, China;; 2 Center for Drug and Clinical Research, Nanfang Hospital, Southern Medical University, Guangzhou, China;; 3 Department of Medical Imaging Center, Nanfang Hospital, Southern Medical University, No. 1838, Guangzhou Avenue North, 510515 Guangzhou, China;; 4 Guangdong Key Laboratory of Liver Disease Research, the 3rd Affiliated Hospital of Sun Yat-sen University, Guangzhou, 510630, China;; 5 Department of Gastric Surgery, Sun Yat-sen University Cancer Center, 651 Dongfeng Road East, Guangzhou, P. R. China, 510060;; 6 State Key Laboratory of Oncology in South China, Collaborative Innovation Center for Cancer Medicine, Guangzhou, P. R. China, 510060;; 7 Department of Gastrointestinal Surgery of the First Affiliated Hospital, Sun Yat-sen University, Guangzhou, 510700, Guangdong, China.

**Keywords:** gastric cancer, age, lymph node positivity

## Abstract

**Object:** The risk of lymph node positivity (LN+) in gastric cancer (GC) impacts therapeutic recommendations. The aim of this study was to quantify the effect of younger age on LN+.

**Methods:** Data from a Chinese multi-institutional database and the US SEER database on stage I to III resected GC were analyzed for the relationship between age and LN+ status. The association of age and LN+ status was examined with logistic regression separately for each T stage, adjusting for multiple covariates. Poisson regression was used to evaluate age and number of LN+.

**Results:** 4,905 and 14,877 patients were identified in the China and SEER datasets respectively. 479 (9.8%) patients were under age 40 years, with 768 (15.7%) between age 40 and 49 years in China dataset, and 416 (2.8%) patients were under age 40 years, with 1176 (7.9%) between age 40 and 49 years in SEER dataset. Both datasets exhibited significantly proportional decreases of N3a and N3b LN+ with age increasing. Patients younger than age 40 years were more likely to show LN+ compared with the reference age 60 to 69 years. The youngest patients had the highest ORs of N1, N2, N3a, and N3b vs N0 LN+ within T4 stage of China dataset and T3 stage of SEER dataset, the values of ORs decreased with increasing age. Young age was a predictor of an increased number of LNs positive for each T stage.

**Conclusion:** In the two large datasets, young age at diagnosis is associated with an increased risk of LN+.

## Introduction

Gastric cancer (GC) is the fifth most commonly diagnosed cancer in the world^1^, with a peak documented incidence among individuals 50-70 years of age^2^, ^3^ and a rare reported incidence among patients 40 years of age or younger^4^-^6^ Despite a gradual decrease in the incidence of GC during the last five decades worldwide, but reverse trends are observed among the young generation in both Western and Eastern populations^2^, ^4^.

The prognostic importance of nodal positivity is reflected in the TNM staging system of gastric cancer^7^. Treatment planning is guided by predicted nodal metastases and prognosis guided by the number of pathologically positive LNs and subsequent accurate staging of the disease. In our clinical practice, we noted that young patients with GC presented a higher rate of lymph node positivity (LN+), particularly those with ≥ 7 LN+. Some studies have shown this trend in breast cancer, and colorectal cancer^8^-^10^. In contrast, LN+ occurring in GC by age groups is a poorly studied clinical entity.

In this study, we performed analyses of two large databases that include various regions, ethnicities, and clinical preferences, which may more accurately portray real-world conditions to confirm the relationship between age and lymph node involvement, while adjusting for other clinicopathologic variables.

## Methods

### Patient Selection

To develop an international dataset with both East Asian and Western gastric cancer patients, data were obtained from 3 cancer centers in China (Nanfang Hospital of Southern Medical University (Guangzhou, China), First Affiliated Hospital of Sun Yat-sen University (Guangzhou, China), and Sun Yat-sen University Cancer Center (Guangzhou, China)) and combined with data from the National Cancer Institute's Surveillance, Epidemiology, and End Results (SEER) database. This study was approved by the research ethics committees at the 3 participating centers, and the informed consent requirement was waved.

The following factors were obtained from the data: age, sex, ethnicity, histology, surgery performed, T-classification, N-classification, total number of LNs examined, and total number of positive LNs. The China dataset from 3 cancer centers is prospectively maintained and all information was pulled directly from the database after meticulous verification through internal quality-control measures. All staging data within the database were updated and coded to confirm to the American Joint Committee on Cancer (AJCC) TNM 7^th^ edition staging system.

Considering the changes in coding and AJCC TNM staging, and to match the time span of the Chinese dataset, we only extracted the data of the years 2000 to 2014 for use from the SEER database. The SEER database was searched, identifying ICD-0-3 site recode for “stomach” and then further narrowed down using the behavior code “malignant”. The ICD-0-3 histology/behavior codes were then used to identify only the cases of gastric adenocarcinoma, eliminating other gastric tumors (neuroendocrine, gastrointestinal stromal tumor, unknown, metastatic disease). Patients who received radiotherapy and/or chemotherapy prior to surgery were excluded, to eliminate the effect of preoperative radiation and chemotherapy on lymph node harvest and positivity. Pathologically staged patients with nonmetastatic adenocarcinoma over the age of 18 years were included. Only patients with at least one lymph node examined were included. All patients were required to have a standard gastric cancer operation, based on the SEER coded description of surgical procedure. Local excision or local destruction procedures were excluded because of the lack of expectation of obtaining lymph nodes with this type of procedure. The following groups of patients were eliminated: patients younger than 18 years of age, patients with multiple primary tumors, and patients with unknown T stage, surgery type, or number of lymph nodes positive.

Patient demographics (race, sex, age at diagnosis, year of diagnosis), tumor characteristics (histologic grade, extension of primary tumor invasion, number of /lymph nodes examined and positive for metastatic disease), and type of surgery were included. T stage, categorized as T1, T2, T3, and T4, was determined by AJCC TNM 7th edition T-classification with “derived ajcc m, 6th ed (2004+)” being recoded accordingly to represent the appropriate depth. Type of surgery performed was divided into total gastrectomy and subtotal gastrectomy, using “rx sum-surg prim site (1998+)” to determine this. If exact surgery could not be determined and fit into these categories, the patient was removed from the dataset along with any patient who did not receive a gastrectomy.

### Outcome Measures and Statistical Analysis

The primary study outcome was LN+ status. Based on the pathological N stage of the 7th edition of AJCC/UICC TNM classification, LN+ status was assigned to one of the four outcome categories: no lymph nodes metastasis (N0 stage, reference category); 1-2 lymph nodes metastasis (N1 stage); 3-6 lymph nodes metastasis (N2 stage); 7-15 lymph nodes metastasis (N3a stage); or ≥16 lymph nodes metastasis (N3b stage). Hence, the outcome of this study was multinomial. Covariates included age, number of LNs examined (LNE), year of diagnosis, type of surgery, tumor grade, sex, and race. Age was included as a categorical variable using 10-year intervals, except for ages 18 to 39 years, because of the smaller number of cases. All analyses were stratified by T stage. Differences in patient characteristics by age were determined using Chi-square tests. Differences in the number of LNE among age groups were compared using one-way analysis of variance (ANOVA). Trends in LN+ with age were evaluated with Cochran Armitage trend tests. In univariate logistic regression analyses with LN+ status as the outcome, the number of LNE was most predictive of LN+ as a log-transformed variable, and therefore was utilized in such a way in multivariable analyses (MVAs). However, results were similar when included as a linear or categorical variable. Logistic regression MVAs were performed for each T stage, with LN+ status as the outcome and age (10-20 year intervals, as above), number of LNE (log transformed), year of diagnosis (in 3-5 year intervals), type of surgery (2 categories), grade (I, II, III, IV, or unknown, in SEER dataset)/ differentiation (well, moderate, poor or undifferentiated, in China dataset), sex, and race (white, black, other) as covariates. Results are represented in the form of odds ratios (ORs) with 95% confidence intervals (CIs) using age 60 to 69 years as the reference category.

In a secondary analysis, we examined the relationship of age at diagnosis and number of positive LNs in those who were node positive. We assessed whether age was associated with the number of positive LNs, using Poisson regression. Results of the Poisson regressions are presented as rate ratios (RRs), again using age 60 to 69 years as the reference category. This provides an adjusted estimate of the ratio of the mean number of positive LNs in a specified age group relative to the age 60 to 69 years age group. For covariate adjustment, we considered the same variables as in the logistic regression models. Because of small cell sizes, tumor grade was collapsed into three categories: I, II, and III/IV/unknown in SEER dataset. All statistical tests were conducted using SPSS version 21.0 (IBM) and R version 3.4.0 (http://www.r-project.org). Statistical significance was set at 2-sided *P* < .05.

## Results

### Descriptive Statistics

We identified 4905 and 14877 patients who met eligibility criteria in the China and SEER datasets respectively. Overall, in China dataset, N0 stage (no LN+) occurred in 1901 (38.8%), N1 (1-2 LN+) occurred in 751 (15.3%) patients, N2 (3-6 LN+) in 899 (18.3%), N3a (7-15 LN+) in 865 (17.6%), and N3b (>15 LN+) in 489 (10.0%) patients. In SEER dataset, N0 stage occurred in 4312 (29.0%), N1 occurred in 3376 (22.7%) patients, N2 in 3191 (21.4%), N3a in 2849 (19.2%), and N3b in 1149 (7.7%) patients. **Table S1-2** summarizes the patient, tumor, and surgical characteristics in the China and SEER dataset respectively. Only 479 (9.8%) of patients were under age 40 years, with 768 (15.7%) between age 40 and 49 years and 1490 (30.4%) between age 50 and 59 years in the China dataset. Only 416 (2.8%) of patients were under age 40 years, with 1176 (7.9%) between age 40 and 49 years and 2446 (16.4%) between age 50 and 59 years in the SEER dataset. The greatest proportion was T4 (58.8%) in the China dataset, whereas the greatest was T3 (43.7%) in the SEER dataset.

Within each T stage, the mean LNE significantly decreased with increasing age in the SEER dataset (**Table 1**). However, only within T3 stage, the mean LNE decreased with increasing age in the China dataset. In total patients, only N3a and N3b LN+ rates obviously decreased with increasing age in China dataset (*P*_trend_ < 0.05, **Table 2**). The youngest patients had the highest LN+ rates. Subgroup analysis by T stage showed that this tendency appeared in each T stage. In SEER dataset, N2, N3a and N3b LN+ rates significantly decreased with increasing age in total patients (*P*_trend_ < 0.001, **Table S3**), and this phenomenon mainly appeared in T3 stage. In univariate logistic analyses of China dataset, the N3b LN+ rates for age 18 to 39 years were statistically significantly higher than patients age 60 to 69 years (reference group) for stages T2, T3, and T4 (**Table 3**), and the N3a LN+ rates were statistically significantly higher for stages T1, T3, and T4. In SEER dataset, the N3b LN+ rates for age 18 to 39 years were statistically significantly higher than patients age 60 to 69 years for stages T2, and T3 (**Table S4**), and the N3a LN+ rates were statistically significantly higher for stage T3.

### Multivariable Analysis of Lymph Node Positivity

We used multivariable logistic regression to examine whether the association between age at diagnosis and LN+ stage was independent of other known risk factors. The adjusted model included number of LNE (log transformed), year of diagnosis, surgery type, grade, sex, and race in SEER dataset. The race was not used to adjust in China dataset, because all patients were the Han nationality. With these covariates, age remained a statistically significant predictor of N3b LN+ for each T stage (**Table 3**), and statistically significant predictor of N3a LN+ for stages T1, T3, T4 in China dataset. Patients younger than age 40 years when diagnosed were more likely to show LN+ compared with the reference age 60 to 69 years, with adjusted ORs of N3b vs N0 LN+ for age 18 to 39 years vs age 60 to 69 years: T1 (OR = 12.039 (95%CI 1.365-106.2), *P* = 0.025), T2 (12.616 (2.091-7 6.134), *P* = 0.006), T3 (3.703 (1.299-10.55), *P* = 0.014), and T4 (2.267 (1.393-3.690), *P* = 0.001), and ORs of N3a vs N0 LN+ for age 18 to 39 years vs age 60 to 69 years: T1 (3.532 (1.199-10.41)), *P* = 0.022), T2 (1.453 (0.449- 4.704), *P* = 0.533), T3 (2.550 (1.083-6.002), *P* = 0.032), and T4 (1.631 (1.066-2.495), *P* = 0.024) (**Table S5-8**). The youngest patients had the highest ORs of N1, N2, N3a, and N3b vs N0 LN+ within T4 stage, the values of ORs decreased with increasing age (**Table 3**). Within T1, T2, and T3 stages, the youngest patients had the highest ORs of N3a vs N0 and N3b vs N0 LN+.

In SEER dataset, with these covariates, age remained a statistically significant predictor of N3a LN+ for T3, and N3b LN+ for T2 stage (**Table S4**). Patients younger than age 40 years when diagnosed were more likely to show LN+ compared with the reference age 60 to 69 years, with adjusted ORs of N3b vs N0 LN+ for age 18 to 39 years vs age 60 to 69 years: T2 (7.390 (1.795-30.42), *P* = 0.006), and ORs of N3a vs N0 LN+ for age 18 to 39 years vs age 60 to 69 years: T3 (2.051(1.287-3.268), *P* = 0.003). The youngest patients had the highest ORs of N1, N2, N3a, and N3b vs N0 LN+ within T3 stage, the values of ORs decreased with increasing age (**Table S4 and 9-12**). Within T2 stage, the youngest patients had the highest ORs of N3b vs N0 LN+, the values of ORs decreased with increasing age.

We further examined the impact of age by looking at the number of positive LNs in node-positive patients. **Table 4 and 5** shows the mean number of positive LNs by age group within T stage in China and SEER datasets. In China dataset, the average number of positive LNs was highest in the youngest age group for each T stage (**Table 4)**, and significantly higher than other age years group. In SEER dataset, the average number of positive LNs was also highest in the youngest age group for stages T1, T2, and T3 (**Table 5**), and the average number of positive LNs decreases with the patients getting older within T2 and T3 stages. In multivariable Poisson analyses of China dataset, adjusting for number of LNE (log transformed) and other covariates, the youngest patients had the highest RRs of positive LNs within each T stage (**Table 4**), with adjusted RRs for age 18 to 39 years vs age 60 to 69 years: T1, (RR = 2.979 (2.484-3.572), *P* < 0.0001), T2 (RR = 1.850 (1.555-2.200), *P* < 0.0001), T3 (RR = 1.662 (1.494-1.849), *P* < 0.0001), and T4 (RR = 1.341 (1.278-1.407), *P* < 0.0001) (**Table S13**). In multivariable Poisson analyses of SEER dataset, the youngest patients had the highest RRs of positive LNs within each T stage except T4 (**Table 5**), with adjusted RRs for age 18 to 39 years vs age 60 to 69 years: T1 (RR = 1.341 (1.010-1.782), *P* = 0.0043), T2 (1.771 (1.073-2.923), *P* = 0.0025), T3 (1.111 (1.042- 1.185), *P* = 0.0013, and T4 (1.239 (1.161-1.323), *P*< 0.0001) (**Table S14-15**). The adjusted mean number of positive LNs for age 18 to 39 years (T4: 2.662±0.345; T2: 4.811±0.689; T3: 8.095±0.937; T4: 9.138±0.565) was higher compared with the 60 to 69 years (T1: 0.818±0.200; T2: 2.380±0.380; T3: 4.609±0.548; T4: 6.611±0.309) age group for each T stage in China dataset. In SEER dataset, the adjusted mean number of positive LNs for age 18 to 39 years was also higher compared with the 60 to 69 years age group for each T stage except T4.

In addition, we observed that after the age of 70 years, increasing age may increase the risk of lymph node positive, with the values of ORs and RRs increased and greater than 1 in some circumstances, such as N3a and N3b vs N0 LN+ for T1 and T4 stages in China and SEER datasets.

## Discussion

Multiple factors are well known to influence the risk of LN+ in gastric cancer patients, such as T stage and histologic grade. However, we are unaware of any previous studies examining LN+ as a function of age in gastric cancer patients. We undertook this study to investigate this question.

Our study showed that younger patients have an increased risk of lymph node metastasis when examined within T stage cohorts, especially for N3a and N3b LN+. This finding persists on multivariable analyses, including potential covariates such as those listed above. As Takatsu et al. showed that the patient proportion having 7 or more lymph node metastases was higher in the young group (under 40 years, 25 %) than in the control group (60-69 years, 16 %)^4^. As the analysis adjusts for an increased number of lymph nodes examined in SEER dataset and T3 stage of China dataset, this is not simply a potential function of younger patients with more easily identifiable nodal tissue. These findings lend more support to the hypothesis that younger GC patients may have an increased predisposition for nodal metastasis.

One possible explanation for our results is that there is a biological difference in the GCs of younger patients that is either because of genetic differences in the tumor or the host. As indicated in previous studies, undifferentiated and diffusely infiltrative GC, and Borrmann types 3 and 4 GC might be more likely to occur in young patients^4^, ^5^, ^11^. Gastric cancer has a more aggressive biologic behavior in young patients than in middle-aged patients and may be associated with a worse prognosis^12^, ^13^. However, the concept of gastric cancer having a poorer prognosis in relatively young patients remains controversial^14^, ^15^. In addition, aging is accompanied by many physiological changes including those in the immune system and elder patients have weaker immune function in contrast with younger individuals^16^-^18^. Several studies supported that there are age-dependent variations on cancer immune surveillance, including degenerative changes of lymph nodes and reduced lymphatic flow to nodes and nodal involution 16, 19-21. These data suggest that GC in younger patients may behave biologically more aggressively than in older patients and fit with our findings.

We also observed that after the age of 70 years, increasing age may increase the risk of lymph node positive, with the values of ORs and RRs increased and greater than 1 (reference, 60-69 years) in some special cases. The similar phenomenon also was observed in breast cancer that the age of 70 years was the turning point for the risk of lymph node involvement^22^. Moreover, 70 years is a clinically relevant turning point, but the biologic reason is not clear^22^, ^23^. Besides, there were some subtle differences in the results between the China and SEER datasets. In the logistic and Poisson regression analysis of T4 stage, the youngest patients had the highest ORs and RRs in China dataset, but this trend was not obvious in SEER dataset. The potential reasons were patients in China presenting with different tumor features, such as more stage 3 disease, more T4 tumors, more distal tumors and more LN metastases^24^.

Clinically, accurate prediction of lymph node involvement is essential in helping doctors make decisions more reasonably^20^, ^25^-^29^. Treatment recommendations for a patient with GC would vary depending on lymph node status. For example, neoadjuvant chemoradiotherapy would only be recommended for II/III stage patients with lymph node metastasis^30^, ^31^. Underestimating the risk for LN+ would lead to lack of neoadjuvant chemotherapy, followed by an increased rate of recurrence and toxicity^32^. In view of the higher rate of N3a and N3b LN+ in younger patients, we suggest that young GC patients receive a thorough and professional assessment of lymph node-bearing regions before surgical treatment.

This study is limited by its retrospective nature. The SEER database is a precious resource that records a great deal of patients' data, but the possibility exists that some of these data have been miscoded. However, this miscoding could be expected to be random and not introduce any systematic bias. In addition, the Lauren classification was not recorded and analysed, which was an important pathological characteristic and associated with lymph node metastasis^33^, ^34^. In a large series of resected EGC from the Italian Research Group for Gastric Cancer (GIRCG) database, submucosal invasion, Lauren diffuse/mixed type, Kodama Pen A type and tumor size were found to be associated with an increased risk of lymph node metastases 35. The risk of positive nodes is particularly high in diffuse-mixed type, an aggressive form of GC with special propensity to lymph node metastasis in advanced GCs 34. The patients' surgery varied depending on the tumor location and other clinicopathological factors, in addition to the experience and judgment of each patient's doctor. These were accounted for in the multivariable analysis, but it is possible that the clinical approach to a young patient may be more aggressive than one who is older, introducing systematic bias. Furthermore, some important information was not assessed in this study, such as the extent of lymphadenectomy, tumor size and location. The assessment of N stage is deeply influenced by the extent of lymphadenectomy (D1 vs. D2). As a result, a “stage migration” can occur especially when a limited lymphadenectomy is performed 33.

In conclusion, our study demonstrates increased risks of LN+ in younger patients with GC, especially for N3a and N3b LN+, after accounting for other known predictive factors. Young patients who have LN+ have higher lymph node ratios. These findings warrant further investigation and could impact the aggressiveness of nodal staging in younger patients with GC.

## Supplementary Material

Supplementary figures and tables.

## Figures and Tables

**Table 1:** Number of LN examined by age and T stage

Age, years	T1				T2				T3				T4		
	N	Mean LNE	SE		N	Mean LNE	SE		N	Mean LNE	SE		N	Mean LNE	SE
**China dataset**															
All	905	29.632	0.649		486	28.802	0.766		628	32.309	0.787		2886	30.406	0.366
18-39	97	29.381	1.955		45	28.956	2.363		70	36.657	2.534		267	30.491	1.135
40-49	159	29.145	1.451		77	34.091	1.940		81	33.025	1.997		451	31.188	0.938
50-59	258	30.946	1.261		153	27.301	1.337		187	33.920	1.484		892	30.482	0.633
60-69	286	29.972	1.204		147	27.776	1.488		199	31.090	1.411		872	30.731	0.707
70+	105	26.448	1.679		64	28.281	1.828		91	27.681	1.845		404	28.611	0.939
P*		0.385				0.052				0.032				0.362	
**SEER dataset**															
All	1002	17.329	0.367		2433	16.320	0.253		6503	18.159	0.156		4939	18.380	0.183
18-39	19	21.158	3.494		48	19.875	1.812		168	20.179	0.996		181	20.624	0.948
40-49	63	20.000	1.507		158	18.500	1.122		519	19.561	0.587		436	19.773	0.599
50-59	158	18.063	1.047		373	18.067	0.702		1066	20.658	0.415		849	19.815	0.463
60-69	272	17.857	0.642		593	17.678	0.511		1616	18.890	0.321		1173	19.199	0.390
70-79	328	17.034	0.625		740	16.085	0.457		1875	17.300	0.282		1370	18.153	0.356
80+	162	14.840	0.890		521	12.868	0.453		1259	15.536	0.309		930	15.283	0.354
P*		0.014				<0.0001				<0.0001				<0.0001	

*P-value from the one-way ANOVA test. Abbreviations: LNE, lymph node examined; SE, standard error; ANOVA, analysis of variance.

**Table 2:** Lymph node positivity and age within T stage groups in China dataset. Table 2. Lymph node positivity and age within T stage groups in China dataset.

Age, year	No.	N1(%)	N2(%)	N3a(%)	N3b(%)
**Total**					
18-39	479	67(14.0)	85(17.7)	102(21.3)	76(15.9)
40-49	768	121(15.8)	121(15.8)	137(17.8)	85(11.1)
50-59	1490	223(15.0)	262(17.6)	270(18.1)	149(10.0)
60-69	1504	235(15.6)	290(19.3)	251(16.7)	123(8.2)
70+	664	105(15.8)	141(21.2)	105(15.8)	56(8.4)
P*		0.777	0.521	0.037	<0.0001
**T stage T1**					
18-39	97	8(8.2)	13(13.4)	8(8.2)	3(3.1)
40-49	159	22(13.8)	12(7.5)	7(4.4)	1(0.6)
50-59	258	26(10.1)	17(6.6)	2(0.8)	2(0.8)
60-69	286	26(9.1)	15(5.2)	8(2.8)	2(0.7)
70+	105	10(9.5)	9(8.6)	5(4.8)	0(0.0)
P*		0.460	0.145	0.185	0.121
**T stage T2**					
18-39	45	10(22.2)	6(13.3)	5(11.1)	6(13.3)
40-49	77	11(14.3)	9(11.7)	5(6.5)	0(0.0)
50-59	153	21(13.7)	29(19.0)	18(11.8)	3(2.0)
60-69	147	26(17.7)	23(15.6)	14(9.5)	2(1.4)
70+	64	16(25.0)	6(9.4)	5(7.8)	0(0.0)
P*		0.724	0.770	0.809	0.016
**T stage T3**					
18-39	70	12(17.1)	11(15.7)	18(25.7)	12(17.1)
40-49	81	12(14.8)	22(27.2)	11(13.6)	7(8.6)
50-59	187	32(17.1)	35(18.7)	28(15.0)	17(9.1)
60-69	199	38(19.1)	46(23.1)	30(15.1)	12(6.0)
70+	91	17(18.7)	16(17.6)	15(16.5)	6(6.6)
P*		0.828	0.550	0.212	0.039
**T stage T4**					
18-39	267	37(13.9)	55(20.6)	71(26.6)	55(20.6)
40-49	451	76(16.9)	78(17.3)	114(25.3)	77(17.1)
50-59	892	144(16.1)	181(20.3)	222(24.9)	127(14.2)
60-69	872	145(16.6)	206(23.6)	199(22.8)	107(12.3)
70+	404	62(15.3)	110(27.2)	80(19.8)	50(12.4)
P*		0.557	0.449	0.054	0.007

*P_trend_ from Cochran Armitage trend test for lymph node positivity and age within T stage and N stage.

**Table 3:** Association of age and LN positivity in China dataset.

Age, year	N1 *vs*. N0		N2 *vs*. N0		N3a *vs*. N0		N3b *vs*. N0	
	OR (95% CI)	*P*	OR (95% CI)	*P*	OR (95% CI)	*P*	OR (95% CI)	*P*
**Unadjusted**								
**T stage T1**								
18-39	1.112(0.481-2.573)	.803	3.133(1.419-6.917)	.005	3.615(1.307-10.004)	.013	5.423(0.887-33.143)	.067
40-49	1.700(0.924-3.126)	.088	1.607(0.729-3.543)	.240	1.757(0.622-4.964)	.287	1.004(0.090-11.19)	.997
50-59	1.114(0.627-1.978)	.713	1.262(0.615-2.590)	.525	0.278(0.058-1.326)	.108	1.114(0.156-7.976)	.915
**60-69**	**Reference**		**Reference**		**Reference**		**Reference**	
70+	1.116(0.516-2.414)	.781	1.741(0.734-4.131)	.209	1.813(0.577-5.701)	.309	/	/
**T stage T2**								
18-39	1.752(0.720-4.267)	.217	1.188(0.423-3.340)	.743	1.627(0.520-5.095)	.403	13.667(2.548-73.304)	.002
40-49	0.667(0.304-1.464)	.313	0.617(0.265-1.437)	.263	0.563(0.192-1.656)	.297	/	/
50-59	0.808(0.421-1.549)	.520	1.261(0.674-2.360)	.469	1.286(0.600-2.756)	.518	1.500(0.244-9.213)	.662
**60-69**	**Reference**		**Reference**		**Reference**		**Reference**	
70+	1.364(0.655-2.841)	.407	0.578(0.217-1.538)	.273	0.792(0.265-2.360)	.675	/	/
**T stage T3**								
18-39	1.356(0.587-3.130)	.475	1.027(0.442-2.387)	.951	2.576(0.172-5.662)	.018	4.294(1.647-11.199)	.003
40-49	0.795(0.365-1.732)	.563	1.204(0.619-2.343)	.585	0.923(0.409-2.083)	.847	1.468(0.526-4.099)	.463
50-59	0.820(0.464-1.449)	.494	0.741(0.429-1.277)	.280	0.908(0.495-1.668)	.757	1.379(0.616-3.088)	.435
**60-69**	**Reference**		**Reference**		**Reference**		**Reference**	
70+	0.883(0.440-1.769)	.725	0.686(0.343-1.372)	.287	0.986(0.473-2.058)	.971	0.986(0.343-2.838)	.980
**T stage T4**								
18-39	1.120(0.696-1.802)	.642	1.171(0.726-1.801)	.470	1.565(1.037-2.363)	.033	2.255(1.439-3.535)	.000
40-49	1.063(0.740-1.527)	.740	0.768(0.542-1.089)	.139	1.162(0.838-1.612)	.369	1.460(1.004-2.122)	.048
50-59	0.979(0.727-1.319)	.891	0.867(0.659-1.140)	.306	1.100(0.841-1.439)	.486	1.171(0.851-1.610)	.333
**60-69**	**Reference**		**Reference**		**Reference**		**Reference**	
70+	0.901(0.617-1.317)	.591	1.126(0.809-1.566)	.483	0.847(0.597-1.203)	.354	0.985(0.654-1.484)	.942
Adjusted for covariates*								
**T stage T1**								
18-39	1.008(0.427-2.383)	.985	3.204(1.395-7.359)	.006	3.532(1.199-10.41)	.022	12.039(1.365-106.2)	.025
40-49	1.603(0.857-2.999)	.139	1.608(0.714-3.623)	.252	1.533(0.521-4.509)	.437	1.757(0.135-22.80)	.667
50-59	1.086(0.606-1.946)	.781	1.239(0.594-2.583)	.568	0.252(0.052-1.224)	.087	1.441(0.168-12.371)	.739
**60-69**	**Reference**		**Reference**		**Reference**		**Reference**	
70+	1.168(0.529-2.580)	.701	1.737(0.702-4.301)	.233	2.263(0.667-7.683)	.190	/	/
**T stage T2**								
18-39	1.829(0.733-4.567)	.196	1.078(0.371-3.129)	.890	1.453(0.449-4.704)	.533	12.616(2.091-76.134)	.006
40-49	0.667(0.297-1.498)	.327	0.578(0.240-1.393)	.222	0.483(0.159-1.474)	.201	0.000E+00	.998
50-59	0.809(0.418-1.563)	.527	1.293(0.675-2.475)	.438	1.237(0.562-2.723)	.597	1.193(0.182-7.836)	.854
**60-69**	**Reference**		**Reference**		**Reference**		**Reference**	
70+	1.311(0.622-2.763)	.477	0.534(0.194-1.464)	.223	0.703(0.229-2.161)	.539	0.000E+00	.998
**T stage T3**								
18-39	1.500(0.618-3.639)	.370	1.106(0.454-2.695)	.825	2.550(1.083-6.002)	.032	3.703(1.299-10.55)	.014
40-49	0.835(0.375-1.861)	.659	1.222(0.613-2.436)	.569	0.947(0.406-2.210)	.900	1.487(0.507-4.360)	.470
50-59	0.916(0.509-1.647)	.769	0.756(0.433-1.321)	.326	0.877(0.466-1.650)	.684	1.227(0.525-2.869)	.637
**60-69**	**Reference**		**Reference**		**Reference**		**Reference**	
70+	0.909(0.446-1.851)	.792	0.756(0.373-1.533)	.438	1.078(0.503-2.311)	.848	1.270(0.419-3.850)	.673
**T stage T4**								
18-39	1.235(0.760-2.009)	.394	1.215(0.783-1.884)	.385	1.631(1.066-2.495)	.024	2.267(1.393-3.690)	.001
40-49	1.129(0.782-1.629)	.517	0.785(0.552-1.117)	.179	1.146(0.819-1.601)	.426	1.326(0.885-1.988)	.172
50-59	0.999(0.740-1.349)	.994	0.870(0.660-1.148)	.325	1.080(0.821-1.419)	.583	1.160(0.826-1.630)	.392
**60-69**	**Reference**		**Reference**		**Reference**		**Reference**	
70+	0.920(0.628-1.349)	.670	1.149(0.824-1.603)	.412	0.901(0.631-1.287)	.566	1.206(0.777-1.872)	.404

CI = confidence interval; Ref = referent; OR = odds ratio. *Covariates include log (number of lymph nodes examined), year of diagnosis, differentiation, sex, type of surgery.

**Table 4:** Association of age and number of positive LNs in node-positive patients in China dataset.

Age, year	N	Mean	SEM	RR(95% CI)	*P*
**T1 (n=905)**					
18-39	97	2.608	0.624	2.979 (2.484-3.572)	<0.0001
40-49	159	1.126	0.256	1.317 (1.084-1.602)	0.006
50-59	258	0.698	0.169	0.818 (0.674-0.993)	0.042
**60-69**	286	0.836	0.186	**Reference**	
70+	105	0.838	0.193	0.958 (0.746-1.230)	0.737
P*		<0.0001			
**T2 (n=486)**					
18-39	45	4.889	1.079	1.850 (1.555-2.200)	<0.0001
40-49	77	1.429	0.339	0.556 (0.448-0.693)	<0.0001
50-59	153	2.392	0.334	0.977 (0.841-1.134)	0.758
**60-69**	147	2.327	0.441	**Reference**	
70+	64	1.609	0.381	0.639 (0.512-0.798)	<0.0001
P*		0.001			
**T3 (n=628)**					
18-39	70	8.829	1.551	1.662 (1.494-1.849)	<0.0001
40-49	81	4.469	0.663	0.973 (0.860-1.102)	0.667
50-59	187	5.064	0.634	1.058 (0.964-1.134)	0.233
**60-69**	199	4.467	0.460	**Reference**	
70+	91	4.044	0.642	1.015 (0.898-1.147)	0.809
P*		0.001			
**T4 (n=2886)**					
18-39	267	9.449	0.663	1.341 (1.278-1.407)	<0.0001
40-49	451	8.100	0.533	1.132 (1.085-1.180)	<0.0001
50-59	892	7.647	0.335	1.143 (1.104-1.184)	<0.0001
**60-69**	872	6.521	0.280	**Reference**	
70+	404	6.693	0.472	1.100 (1.050-1.152)	<0.0001
P*		<0.0001			

*P-value from the one-way ANOVA test.

**Table 5:** Association of age and number of positive LNs in node-positive patients in SEER dataset.

Age, year	N	Mean	SEM	RR(95% CI)	P
**T1: stratification by Race**					
**White (n=649)**					
18-39	14	3.857	1.292	1.341 (1.010-1.782)	0.043
40-49	34	2.912	0.576	1.050 (0.845-1.304)	0.661
50-59	89	2.326	0.264	0.895 (0.759-1.054)	0.184
**60-69**	189	2.540	0.318	**Reference**	
70-79	216	2.505	0.253	1.032 (0.913-1.168)	0.612
80+	107	3.308	0.443	1.515 (1.318-1.743)	<0.001
P*		0.363			
**T2 (n=2433)**					
18-39	48	3.375	1.051	1.771 (1.073-2.923)	0.025
40-49	158	2.595	0.395	1.250 (0.920-1.698)	0.153
50-59	373	2.123	0.199	1.096 (0.872-1.377)	0.434
**60-69**	593	1.750	0.157	**Reference**	
70-79	740	1.445	0.101	0.645 (0.517-0.806)	0.0001
80+	521	1.511	0.136	0.976 (0.780-1.221)	0.83
P*		<0.0001			
**T3 (n=6503)**					
18-39	168	6.286	0.494	1.111 (1.042-1.185)	0.0013
40-49	519	5.337	0.300	0.972 (0.931-1.015)	0.202
50-59	1066	5.460	0.211	0.989 (0.956-1.022)	0.504
**60-69**	1616	5.033	0.168	**Reference**	
70-79	1875	4.341	0.146	0.949 (0.920-0.979)	0.001
80+	1259	3.770	0.159	0.903 (0.871-0.937)	<0.0001
P*		<0.0001			
**T4 (n=4939)**					
18-39	181	6.773	0.465	1.239 (1.161-1.323)	<0.0001
40-49	436	7.982	0.440	1.225 (1.152-1.302)	<0.0001
50-59	849	7.840	0.302	1.276 (1.202-1.354)	<0.0001
**60-69**	1173	7.812	0.256	**Reference**	
70-79	1370	7.466	0.236	1.296 (1.222-1.376)	<0.0001
80+	930	6.316	0.235	1.293 (1.215-1.375)	<0.0001
P*		<0.0001			

*P-value from the one-way ANOVA test. RR, Rate Ratio.
