# Supplementary material for: Young age increases risk for lymph node positivity in gastric cancer: A Chinese multi-institutional database and US SEER database study
Source: J Cancer. 2020 Jan 1;11(3):678–85. doi: 10.7150/jca.37531 (PMC6959045; doi:10.7150/jca.37531)
Supplement: Supplementary file 1 — Supplementary figures and tables. [file jcav11p0678s1.pdf]

Supplementary Materials

Table S1. Patient and tumor characteristics by age at diagnosis in China dataset.

		Age at diagnosis, y					
		18-39	40-49	50-59	60-69	70+	
**Characteristic**	**No. (%)**	**Percent (within age group)**					*P**
**Age at diagnosis, y**	4905(100%)						
18-39	479(9.8%)						
40-49	768(15.7%)						
50-59	1490(30.4%)						
60-69	1504(30.7%)						
70+	664(13.5%)						
**Sex**							< 0.0001
Female	1545(31.5%)	16.2%	19.0%	27.6%	25.1%	12.1%	
Male	3360(68.5%)	6.8%	14.1%	31.6%	33.2%	14.2%	
**T stage**							0.310
T1	905(18.5%)	10.7%	17.6%	28.5%	31.6%	11.6%	
T2	486(9.9%)	9.3%	15.8%	31.5%	30.2%	13.2%	
T3	628(12.8%)	11.1%	12.9%	29.8%	31.7%	14.5%	
T4	2886(58.8%)	9.3%	15.6%	30.9%	30.2%	14.0%	
**Differentiation**							< 0.0001
Well	274(5.6%)	8.4%	12.4%	29.9%	32.1%	17.2%	
Moderate	1206(24.6%)	6.0%	12.3%	31.1%	34.1%	16.6%	
poor and undifferentiated	3425(69.8%)	11.2%	17.1%	30.2%	29.3%	12.2%	
**Type of gastrectomy**							0.776
Total	3231(65.9%)	9.7%	15.4%	30.7%	30.4%	13.9%	
Subtotal	1674(34.1%)	9.9%	16.2%	29.8%	31.2%	12.9%	

* Characteristics differ by age group, Chi-square tests.

Table S2. Patient and tumor characteristics by age at diagnosis in SEER dataset.*

		Age at diagnosis, y					
		18-39	40-49	50-59	60-69	70-79	80+
**Characteristic**	**No. (%)**	**Percent (within age group)**					
**Age at diagnosis, y**	14877(100%)						
18-39	416(2.8%)						
40-49	1176(7.9%)						
50-59	2446(16.4%)						
60-69	3654(24.6%)						
70-79	4313(29.0%)						
80+	2872(19.3%)						
**Race**							
White	9701(65.2%)	2.8%	7.5%	15.6%	24.2%	29.3%	20.7%
Black	2103(14.1%)	2.8%	9.7%	19.0%	27.2%	27.3%	14.1%
Other	3073(20.7%)	2.9%	7.9%	17.5%	23.9%	29.3%	18.5%
**Sex**							
Female	5719(38.4%)	3.6%	8.6%	15.1%	21.5%	27.9%	23.4%
Male	9158(61.6%)	2.3%	7.5%	17.3%	26.5%	29.7%	16.8%
**T stage**							
T1	1002(6.7%)	1.9%	6.3%	15.8%	27.1%	32.7%	16.2%
T2	2433(16.4%)	2.0%	6.5%	15.3%	24.4%	30.4%	21.4%
T3	6503(43.7%)	2.6%	8.0%	16.4%	24.9%	28.8%	19.4%
T4	4939(33.2%)	3.7%	8.8%	17.2%	23.7%	27.7%	18.8%
**Grade**							
I	418(2.8%)	1.0%	6.5%	15.6%	22.7%	34.9%	19.4%
II	3560(23.9%)	1.3%	4.5%	12.8%	24.5%	32.9%	24.0%
III	10032(67.4%)	3.3%	9.0%	17.4%	24.6%	27.7%	18.0%
IV	407(2.7%)	3.7%	9.8%	17.2%	24.1%	27.3%	17.9%
Unknown	460(3.1%)	3.0%	9.8%	23.5%	27.0%	23.5%	13.3%
**Type of gastrectomy**							
Total	3559(23.9%)	3.6%	9.7%	19.4%	26.2%	27.3%	13.8%
Subtotal	11318(76.1%)	2.5%	7.3%	15.5%	24.1%	29.5%	21.0%

* Characteristics differ by age group, Chi-square tests; all $P < .0001$.

Table S3. Lymph node positivity and age within T stage groups in SEER dataset.

Age, year	No.	N1(%)	N2(%)	N3a(%)	N3b(%)
**Total**					
18-39	416	82(19.7)	96(23.1)	111(26.7)	41(9.9)
40-49	1176	246(20.9)	250(21.3)	266(22.6)	119(10.1)
50-59	2446	516(21.1)	542(22.2)	547(22.4)	207(8.5)
60-69	3654	796(21.8)	824(22.6)	709(19.4)	300(8.2)
70-79	4313	1020(23.6)	896(20.8)	751(17.4)	316(7.3)
80+	2872	716(24.9)	583(20.3)	465(16.2)	166(5.8)
*P**		0.361	<0.0001	<0.0001	<0.0001
**T stage T1**					
18-39	19	7(36.8)	5(26.3)	4(21.1)	0(0.0)
40-49	63	29(46.0)	15(23.8)	9(14.3)	2(3.2)
50-59	158	93(58.9)	32(20.3)	11(7.0)	1(0.6)
60-69	272	135(49.6)	69(25.4)	15(5.5)	5(1.8)
70-79	328	184(56.1)	75(22.9)	16(4.9)	6(1.8)
80+	162	96(59.3)	35(21.6)	15(9.3)	3(1.9)
*P**		0.36	0.639	0.692	0.658
**T stage T2**					
18-39	48	10(20.8)	6(12.5)	3(6.3)	4(8.3)
40-49	158	33(20.9)	27(17.1)	12(7.6)	7(4.4)
50-59	373	79(21.2)	63(16.9)	31(8.3)	5(1.3)
60-69	593	134(22.6)	71(12.0)	43(7.3)	7(1.2)
70-79	740	170(23.0)	110(14.9)	31(4.2)	6(0.8)
80+	521	134(25.7)	47(9.0)	34(6.5)	3(0.6)
*P**		0.797	0.094	0.26	0.009
**T stage T3**					
18-39	168	33(19.6)	37(22.0)	50(29.8)	16(9.5)
40-49	519	110(21.2)	102(19.7)	126(24.3)	43(8.3)
50-59	1066	211(19.8)	261(24.5)	244(22.9)	79(7.4)
60-69	1616	347(21.5)	386(23.9)	336(20.8)	106(6.6)
70-79	1875	418(22.3)	420(22.4)	331(17.7)	105(5.6)
80+	1259	284(22.6)	279(22.2)	190(15.1)	54(4.3)
*P**		0.125	0.024	<0.0001	<0.0001
**T stage T4**					
18-39	181	32(17.7)	48(26.5)	54(29.8)	21(11.6)
40-49	436	74(17.0)	106(24.3)	119(27.3)	67(15.4)
50-59	849	133(15.7)	186(21.9)	261(30.7)	122(14.4)
60-69	1173	180(15.3)	298(25.4)	315(26.9)	182(15.5)
70-79	1370	248(18.1)	291(21.2)	373(27.2)	199(14.5)
80+	930	202(21.7)	222(23.9)	226(24.3)	106(11.4)
*P**		0.846	0.961	0.387	0.884

*P_{trend} from Cochran Armitage trend test for lymph node positivity and age within T stage and N stage.

Table S4. Association of age and LN positivity in SEER dataset.

Age, year	N1 *vs*. N0		N2 *vs*. N0		N3a *vs*. N0		N3b *vs*. N0	
	HR (95% CI)	*P*	HR (95% CI)	*P*	HR (95% CI)	*P*	HR (95% CI)	*P*
**Unadjusted**								
**T stage T1**								
18-39	0.830(0.206-3.338)	.793	1.159(0.264-5.083)	.844	4.267(0.857-21.245)	.076	/	
40-49	1.289(0.551-3.013)	.558	1.304(0.513-3.318)	.577	3.600(1.181-10.978)	.024	2.400(0.396-14.556)	.341
50-59	1.575(0.884-2.803)	.123	1.060(0.547-2.056)	.863	1.676(0.660-4.256)	.277	0.457(0.050-4.156)	.487
**60-69**	**Reference**		**Reference**		**Reference**		**Reference**	
70-79	1.392(0.879-2.204)	.158	1.110(0.661-1.864)	.693	1.089(0.484-2.452)	.836	1.226(0.350-4.291)	.750
80+	2.626(1.349-5.112)	.005	1.873(0.898-3.908)	.094	3.692(1.439-9.473)	.007	2.215(0.467-10.51)	.317
**T stage T2**								
18-39	1.009(0.472-2.158)	.982	1.143(0.708-2.359)	.778	0.943(0.273-3.256)	.926	7.726(2.118-28.18)	.002
40-49	1.054(0.670-1.657)	.821	1.627(0.981-2.700)	.060	1.194(0.602-2.369)	.612	4.278(1.459-12.55)	.008
50-59	1.022(0.735-1.420)	.897	1.538(1.049-2.254)	.027	1.250(0.762-2.049)	.377	1.238(0.388-3.954)	.718
**60-69**	**Reference**		**Reference**		**Reference**		**Reference**	
70-79	1.014(0.776-1.325)	.921	1.238(0.889-1.723)	.206	0.576(0.355-0.934)	.025	0.685(0.228-2.057)	.500
80+	1.116(0.839-1.484)	.453	0.738(0.495-1.101)	.137	0.882(0.548-1.419)	.605	0.478(0.123-1.865)	.288
**T stage T3**								
18-39	1.311(0.790-2.174)	.295	1.321(0.807-2.162)	.268	2.051(1.287-3.268)	.003	2.080(1.101-3.931)	.024
40-49	1.013(0.760-1.350)	.930	0.844(0.632-1.129)	.253	1.198(0.906-1.586)	.205	1.296(0.867-1.939)	.206
50-59	0.990(0.787-1.243)	.928	1.100(0.885-1.368)	.390	1.182(0.945-1.478)	.144	1.213(0.873-1.684)	.249
**60-69**	**Reference**		**Reference**		**Reference**		**Reference**	
70-79	0.884(0.732-1.067)	.198	0.798(0.664-0.961)	.017	0.723(0.595-0.879)	.001	0.727(0.540-0.978)	.035
80+	0.799(0.651-0.980)	.031	0.705(0.576-0.863)	.001	0.552(0.442-0.688)	.000	0.497(0.349-0.707)	.000
**T stage T4**								
18-39	1.354(0.777-2.359)	.285	1.227(0.737-2.043)	.432	1.305(0.791-2.153)	.296	0.879(0.478-1.616)	.677
40-49	1.163(0.792-1.707)	.441	1.006(0.708-2.359)	.973	1.069(0.757-1.508)	.706	1.041(0.704-1.539)	.839
50-59	0.995(0.730-1.356)	.976	0.841(0.635-1.114)	.227	1.116(0.853-1.461)	.424	0.903(0.660--1.235)	.523
**60-69**	**Reference**		**Reference**		**Reference**		**Reference**	
70-79	1.053(0.807-1.375)	.703	0.747(0.584-0.954)	.020	0.905(0.713-1.149)	.413	0.836(0.636-1.099)	.198
80+	1.277(0.959-1.700)	.094	0.848(0.649-1.108)	.226	0.816(0.626-1.065)	.135	0.663(0.484-0.908)	.010
**Adjusted for covariates***								
**T stage T1**								
18-39	1.279(0.222-7.365)	.783	1.872(0.290-12.070)	.510	5.339(0.697-40.91)	.107	/	
40-49	1.815(0.660-4.997)	.248	1.834(0.615-5.465)	.276	4.987(1.402-17.74)	.013	3.824(0.551-28.63)	.192
50-59	1.356(0.655-2.809)	.412	0.869(0.387-1.950)	.733	1.357(0.469-3.927)	.573	0.412(0.037-4.584)	.470
**60-69**	**Reference**		**Reference**		**Reference**		**Reference**	
70-79	1.623(0.924-2.851)	.092	1.403(0.756-2.602)	.283	1.423(0.588-3.447)	.434	1.544(0.385-6.190)	.540
80+	2.189(0.977-4.904)	.057	1.948(0.813-4.667)	.135	4.729(1.634-13.69)	.004	4.510(0.779-26.114)	.093
**T stage T2**								
18-39	0.976(0.453-2.102)	.950	1.048(0.407-2.697)	.923	0.730(0.206-2.591)	.626	7.390(1.795-30.42)	.006
40-49	1.023(0.647-1.616)	.922	1.516(0.904-2.542)	.115	1.121(0.554-2.270)	.751	2.599(0.789-8.563)	.117
50-59	1.033(0.741-1.439)	.849	1.523(1.031-2.250)	.035	1.291(0.775-2.153)	.327	0.960(0.283-3.255)	.947
**60-69**	**Reference**		**Reference**		**Reference**		**Reference**	
70-79	1.039(0.793-1.362)	.781	1.347(0.962-1.886)	.083	0.671(0.409-1.100)	.114	0.709(0.226-2.226)	.555

80+	1.178(0.878-1.581)	.275	0.949(0.628-1.432)	.802	1.298(0.789-2.136)	.304	0.882(0.215-3.621)	.861
**T stage T3**								
18-39	1.294(0.778-2.152)	.321	1.255(0.762-2.066)	.372	1.821(1.122-2.956)	.015	1.701(0.841-3.440)	.139
40-49	0.980(0.734-1.308)	.889	0.792(0.590-1.062)	.119	1.074(0.801-1.438)	.634	1.048(0.667-1.647)	.840
50-59	0.978(0.777-1.231)	.849	1.057(0.847-1.319)	.626	1.051(0.833-1.327)	.676	0.937(0.651-1.349)	.725
**60-69**	**Reference**		**Reference**		**Reference**		**Reference**	
70-79	0.889(0.736-1.074)	.224	0.837(0.693-1.009)	.063	0.801(0.654-0.982)	.033	0.927(0.667-1.289)	.652
80+	0.810(0.659-0.997)	.047	0.769(0.625-0.947)	.013	0.666(0.528-0.840)	.001	0.806(0.545-1.193)	.281
**T stage T4**								
18-39	1.361(0.778-2.381)	0.281	1.073(0.640-1.800)	.788	0.988(0.587-1.661)	.962	0.530(0.269-1.043)	.066
40-49	1.177(0.799-1.733)	0.410	0.935(0.654-1.337	.712	0.908(0.632-1.305)	.603	0.839(0.538-1.308)	.439
50-59	0.993(0.727-1.355)	0.962	0.797(0.599-1.061)	.121	0.999(0.752-1.328)	.997	0.764(0.534-1.094)	.142
**60-69**	**Reference**		**Reference**		**Reference**		**Reference**	
70-79	1.075(0.821-1.407)	0.599	0.782(0.609-1.004)	.054	1.017(0.790-1.309)	.899	0.996(0.726-1.367)	.982
80+	1.287(0.961-1.722)	0.090	0.961(0.729-1.266)	.777	1.106(0.832-1.470)	.487	1.164(0.810-1.674)	.411

Table S5. Multivariate logistic analysis of patient and tumor characteristics in T1 stage of China dataset.

	N1 vs N0		N2 vs N0		N3a vs N0		N3b vs N0	
	OR (95%)	*P*	OR (95%)	*P*	OR (95%)	*P*	OR (95%)	*P*
**Number of LN examined (continuous)**								
Log transformed	0.662(0.330-1.327)	0.245	2.795(1.076-7.260)	0.035	4.966(1.190-20.73)	0.028	292.7(6.815-12573)	0.003
**Age**								
18-39 yrs	1.008(0.427-2.383)	0.985	3.204(1.395-7.359)	0.006	3.532(1.199-10.41)	0.022	12.039(1.365-106.2)	0.025
40-49 yrs	1.603(0.857-2.999)	0.139	1.608(0.714-3.623)	0.252	1.533(0.521-4.509)	0.437	1.757(0.135-22.80)	0.667
50-59 yrs	1.086(0.606-1.946)	0.781	1.239(0.594-2.583)	0.568	0.252(0.052-1.224)	0.087	1.441(0.168-12.371)	0.739
**60-69 yrs**	**Reference**		**Reference**		**Reference**		**Reference**	
70+ yrs	1.168(0.529-2.580)	0.701	1.737(0.702-4.301)	0.233	2.263(0.667-7.683)	0.190	/	/
**Gender**								
Male vs Female	0.962(0.601-1.542)	0.874	0.733(0.411-1.305)	0.291	1.241(0.563-2.736)	0.592	0.857(0.152-4.829)	0.861
**Differentiation**								
Moderate vs well	1.444(0.579-3.605)	0.431	0.639(0.244-1.673)	0.362	0.908(0.140-5.863)	0.919	0.578(0.040-8.302)	0.687
poor and undifferentiated vs well	2.320(1.009-5.333)	0.047	1.263(0.569-2.805)	0.566	3.266(0.698-15.28)	0.133	0.518(0.049-5.443)	0.583
**Type of gastrectomy**								
Subtotal vs Total	0.909(0.532-1.556)	0.729	0.937(0.496-1.771)	0.842	0.874(0.364-2.100)	0.764	0.123(0.021-0.729)	0.021
**Year of Diagnosis**								
2006-07 vs 2004-05	0.593(0.216-1.624)	0.309	0.438(0.147-1.308)	0.139	0.106(0.012-0.895)	0.039	0.922(0.075-11.326)	0.949
2008-09 vs 2004-05	0.658(0.306-1.412)	0.282	0.283(0.114-0.708)	0.007	0.118(0.029-0.485)	0.003	/	/
2010-11 vs 2004-05	0.641(0.302-1.361)	0.247	0.520(0.239-1.130)	0.099	0.123(0.031-0.486)	0.003	0.271(0.022-3.282)	0.305
2012-14 vs 2004-05	0.464(0.233-0.926)	0.029	0.141(0.062-0.317)	0.000	0.101(0.035-0.290)	0.000	0.033(0.004-0.290)	0.002

Table S6. Multivariate logistic analysis of patient and tumor characteristics in T2 stage of China dataset.

	N1 vs N0		N2 vs N0		N3a vs N0		N3b vs N0	
	OR(95%)	*P*	OR(95%)	*P*	OR(95%)	*P*	OR(95%)	*P*
**Number of LN examined (continuous)**								
Log transformed	1.720(0.679-4.355)	0.252	3.750(1.311-10.725)	0.014	4.218(1.146-15.52)	0.030	15.38(0.422-560.2)	0.136
**Age**								
18-39 yrs	1.829(0.733-4.567)	0.196	1.078(0.371-3.129)	0.890	1.453(0.449-4.704)	0.533	12.616(2.091-76.134)	0.006
40-49 yrs	0.667(0.297-1.498)	0.327	0.578(0.240-1.393)	0.222	0.483(0.159-1.474)	0.201	0.000	0.998
50-59 yrs	0.809(0.418-1.563)	0.527	1.293(0.675-2.475)	0.438	1.237(0.562-2.723)	0.597	1.193(0.182-7.836)	0.854
**60-69 yrs**	**Reference**		**Reference**		**Reference**		**Reference**	
70+ yrs	1.311(0.622-2.763)	0.477	0.534(0.194-1.464)	0.223	0.703(0.229-2.161)	0.539	0.000	0.998
**Gender**								
Male vs Female	0.905(0.503-1.629)	0.740	1.816(1.018-3.237)	0.043	2.044(1.048-3.985)	0.036	1.444(0.362-5.763)	0.603
**Differentiation**								
Moderate vs well	1.677(0.436-6.446)	0.452	3.075(0.632-14.96)	0.164	0.564(0.143-2.221)	0.413	0.115(0.008-1.634)	0.110
poor and undifferentiated vs well	1.734(0.471-6.389)	0.408	2.751(0.587-12.89)	0.199	1.048(0.312-3.524)	0.939	0.294(0.038-2.241)	0.237
**Type of gastrectomy**								
Subtotal vs Total	1.453(0.781-2.705)	0.238	2.084(1.030-4.217)	0.041	1.987(0.858-4.606)	0.109	0.903(0.182-4.483)	0.900
**Year of Diagnosis**								
2006-07 vs 2004-05	0.913(0.223-3.730)	0.899	0.247(0.070-0.874)	0.030	0.395(0.085-1.838)	0.237	0.765	1.000
2008-09 vs 2004-05	0.999(0.271-3.675)	0.998	0.239(0.078-0.729)	0.012	0.240(0.056-1.034)	0.056	51950150.525	0.000
2010-11 vs 2004-05	1.345(0.371-4.866)	0.652	0.265(0.087-0.808)	0.020	0.442(0.112-1.749)	0.244	58662579.093	0.000
2012-14 vs 2004-05	0.657(0.174-2.479)	0.535	0.172(0.056-0.537)	0.002	0.231(0.055-0.964)	0.044	10148106.883	0.000

Table S7. Multivariate logistic analysis of patient and tumor characteristics in T3 stage of China dataset.

	N1 vs N0		N2 vs N0		N3a vs N0		N3b vs N0	
	OR(95%)	*P*	OR(95%)	*P*	OR(95%)	*P*	OR(95%)	*P*
**Number of LN examined (continuous)**								
Log transformed	0.433(0.193-0.969)	0.042	1.660(0.690-3.995)	0.258	1.938(0.712-5.275)	0.195	41.796(8.096-215.8)	0.000
**Age**								
18-39 yrs	1.500(0.618-3.639)	0.370	1.106(0.454-2.695)	0.825	2.550(1.083-6.002)	0.032	3.703(1.299-10.55)	0.014
40-49 yrs	0.835(0.375-1.861)	0.659	1.222(0.613-2.436)	0.569	0.947(0.406-2.210)	0.900	1.487(0.507-4.360)	0.470
50-59 yrs	0.916(0.509-1.647)	0.769	0.756(0.433-1.321)	0.326	0.877(0.466-1.650)	0.684	1.227(0.525-2.869)	0.637
**60-69 yrs**	**Reference**		**Reference**		**Reference**		**Reference**	
70+ yrs	0.909(0.446-1.851)	0.792	0.756(0.373-1.533)	0.438	1.078(0.503-2.311)	0.848	1.270(0.419-3.850)	0.673
**Gender**								
Male vs Female	1.154(0.683-1.948)	0.593	1.343(0.824-2.190)	0.236	1.327(0.777-2.267)	0.300	1.159(0.576-2.333)	0.679
**Differentiation**								
Moderate vs well	0.380(0.084-1.716)	0.208	0.528(0.131-2.131)	0.369	0.372(0.075-1.844)	0.226	0.236(0.048-1.168)	0.077
poor and undifferentiated vs well	0.755(0.175-3.251)	0.706	0.946(0.242-3.696)	0.937	1.160(0.251-5.361)	0.849	0.682(0.151-3.078)	0.619
**Type of gastrectomy**								
Subtotal vs Total	1.263(0.742-2.151)	0.389	0.889(0.554-1.427)	0.626	1.154(0.671-1.983)	0.604	0.693(0.361-1.330)	0.270
**Year of Diagnosis**								
2006-07 vs 2004-05	1.347(0.103-17.69)	0.821	0.000	0.996	999.000	0.000	21505598.239	0.991
2008-09 vs 2004-05	0.683(0.117-3.984)	0.672	3.521(0.485-25.56)	0.213	999.000	0.000	1.309	1.000
2010-11 vs 2004-05	2.724(1.040-7.134)	0.041	8.068(1.724-37.77)	0.008	999.000	0.000	21106556.780	0.991
2012-14 vs 2004-05	1.533(0.582-4.041)	0.387	5.186(1.110-24.23)	0.036	999.000	0.000	6198545.237	0.992

Table S8. Multivariate logistic analysis of patient and tumor characteristics in T4 stage of China dataset.

	N1 vs N0		N2 vs N0		N3a vs N0		N3b vs N0	
	OR (95%)	*P*	OR (95%)	*P*	OR (95%)	*P*	OR (95%)	*P*
**Number of LN examined (continuous)**								
Log transformed	0.692(0.456-1.051)	0.084	0.989(0.669-1.464)	0.958	5.484(3.569-8.425)	0.000	150.6(78.62-288.6)	0.000
**Age**								
18-39 yrs	1.235(0.760-2.009)	0.394	1.215(0.783-1.884)	0.385	1.631(1.066-2.495)	0.024	2.267(1.393-3.690)	0.001
40-49 yrs	1.129(0.782-1.629)	0.517	0.785(0.552-1.117)	0.179	1.146(0.819-1.601)	0.426	1.326(0.885-1.988)	0.172
50-59 yrs	0.999(0.740-1.349)	0.994	0.870(0.660-1.148)	0.325	1.080(0.821-1.419)	0.583	1.160(0.826-1.630)	0.392
**60-69 yrs**	**Reference**		**Reference**		**Reference**		**Reference**	
70+ yrs	0.920(0.628-1.349)	0.670	1.149(0.824-1.603)	0.412	0.901(0.631-1.287)	0.566	1.206(0.777-1.872)	0.404
**Gender**								
Male vs Female	0.734(0.562-0.958)	0.023	0.853(0.671-1.084)	0.193	0.757(0.596-0.960)	0.022	0.965(0.729-1.276)	0.801
**Differentiation**								
Moderate vs well	1.508(0.823-2.763)	0.184	1.336(0.770-2.318)	0.303	1.305(0.727-2.343)	0.372	1.233(0.560-2.715)	0.602
poor and undifferentiated vs well	1.205(0.675-2.151)	0.528	1.155(0.683-1.952)	0.591	1.783(1.026-3.100)	0.040	2.463(1.168-5.195)	0.018
**Type of gastrectomy**								
Subtotal vs Total	0.855(0.665-1.100)	0.224	0.789(0.627-0.994)	0.044	0.842(0.670-1.057)	0.138	0.560(0.429-0.732)	0.000
**Year of Diagnosis**								
2006-07 vs 2004-05	1.287(0.788-2.102)	0.314	1.310(0.828-2.075)	0.249	1.236(0.756-2.020)	0.397	1.051(0.563-1.961)	0.876
2008-09 vs 2004-05	0.910(0.605-1.370)	0.651	0.936(0.641-1.368)	0.733	1.069(0.719-1.590)	0.742	0.752(0.452-1.253)	0.274
2010-11 vs 2004-05	1.037(0.671-1.602)	0.871	1.060(0.709-1.584)	0.777	1.135(0.746-1.725)	0.554	1.106(0.659-1.856)	0.702
2012-14 vs 2004-05	0.810(0.527-1.244)	0.335	0.720(0.483-1.072)	0.105	0.610(0.404-0.922)	0.019	0.337(0.203-0.558)	0.000

Table S9. Multivariate logistic analysis of patient and tumor characteristics in T1 stage of SEER dataset.

	N1 vs N0		N2 vs N0		N3a vs N0		N3b vs N0	
	OR (95%)	*P*	OR (95%)	*P*	OR (95%)	*P*	OR (95%)	*P*
**Number of LN examined (continuous)**								
Log transformed	2.769(1.268-6.047)	0.011	10.576(4.348-25.726)	0.000	67.782(19.148-239.94)	0.000	6007(292.2-9999)	0.000
**Age**								
18-39 yrs	1.279(0.222-7.365)	0.783	1.872(0.290-12.070)	0.510	5.339(0.697-40.906)	0.107	/	
40-49 yrs	1.815(0.660-4.997)	0.248	1.834(0.615-5.465)	0.276	4.987(1.402-17.739)	0.013	3.824(0.551-28.63)	0.192
50-59 yrs	1.356(0.655-2.809)	0.412	0.869(0.387-1.950)	0.733	1.357(0.469-3.927)	0.573	0.412(0.037-4.584)	0.470
**60-69 yrs**	**Reference**		**Reference**		**Reference**		**Reference**	
70-79 yrs	1.623(0.924-2.851)	0.092	1.403(0.756-2.602)	0.283	1.423(0.588-3.447)	0.434	1.544(0.385-6.190)	0.540
80+ yrs	2.189(0.977-4.904)	0.057	1.948(0.813-4.667)	0.135	4.729(1.634-13.687)	0.004	4.510(0.779-26.114)	0.093
**Race**								
Black vs White	2.701(1.211-6.024)	0.015	4.579(1.973-10.628)	0.000	1.576(0.475-5.226)	0.457	0.000	0.997
Other vs White	2.264(1.257-4.080)	0.007	1.986(1.040-3.793)	0.038	1.876(0.840-4.190)	0.125	1.689(0.441-6.464)	0.444
**Sex**								
Male vs Female	1.920(1.163-3.170)	0.011	1.769(1.024-3.055)	0.041	1.803(0.898-3.621)	0.097	3.111(0.963-10.049)	0.058
**Grade**								
II vs I	2.586(0.470-14.231)	0.275	3.271(0.527-20.291)	0.203	2.608(0.270-25.160)	0.407	7165154.314	0.000
III vs I	0.207(0.044-0.978)	0.047	0.394(0.074-2.098)	0.275	0.406(0.049-3.390)	0.405	8078036.945	
IV vs I	0.304(0.042-2.219)	0.241	0.676(0.076-6.028)	0.726	0.385(0.016-9.089)	0.554	0.415	1.000
unknown vs I	1.188(0.133-10.600)	0.878	0.646(0.052-8.092)	0.735	3.394(0.200-57.63)	0.398	1.674	1.000
**Type of gastrectomy**								
Subtotal vs Total	2.327(1.298-4.172)	0.005	3.055(1.581-5.904)	0.001	3.403(1.417-8.173)	0.006	5.035(0.922-27.51)	0.062
**Year of Diagnosis**								
2006-07 vs 2004-05	0.875	1.000	0.754	1.000	0.812	1.000	0.290	1.000
2008-09 vs 2004-05	0.000	0.991	0.000	0.991	0.000	0.991	0.000	0.990
2010-11 vs 2004-05	0.000	0.991	0.000	0.990	0.000	0.991	0.000	0.991
2012-14 vs 2004-05	0.969	1.000	0.651	1.000	1.008	1.000	1.188	1.000

Table S10. Multivariate logistic analysis of patient and tumor characteristics in T2 stage of SEER dataset.

	N1 vs N0		N2 vs N0		N3a vs N0		N3b vs N0	
	OR (95%)	P	OR (95%)	P	OR (95%)	P	OR (95%)	P
**Number of LN examined (continuous)**								
Log transformed	1.333(1.003-1.772)	0.048	3.361(2.254-5.012)	0.000	11.138(5.990-20.708)	0.000	518(94.47-2842)	0.000
**Age**								
18-39 yrs	0.976(0.453-2.102)	0.950	1.048(0.407-2.697)	0.923	0.730(0.206-2.591)	0.626	7.390(1.795-30.424)	0.006
40-49 yrs	1.023(0.647-1.616)	0.922	1.516(0.904-2.542)	0.115	1.121(0.554-2.270)	0.751	2.599(0.789-8.563)	0.117
50-59 yrs	1.033(0.741-1.439)	0.849	1.523(1.031-2.250)	0.035	1.291(0.775-2.153)	0.327	0.960(0.283-3.255)	0.947
**60-69 yrs**	**Reference**		**Reference**		**Reference**		**Reference**	
70-79 yrs	1.039(0.793-1.362)	0.781	1.347(0.962-1.886)	0.083	0.671(0.409-1.100)	0.114	0.709(0.226-2.226)	0.555
80+ yrs	1.178(0.878-1.581)	0.275	0.949(0.628-1.432)	0.802	1.298(0.789-2.136)	0.304	0.882(0.215-3.621)	0.861
**Race**								
Black vs White	1.168(0.871-1.565)	0.301	1.352(0.948-1.927)	0.096	1.629(0.998-2.658)	0.051	2.727(1.006-7.391)	0.049
Other vs White	0.821(0.640-1.053)	0.121	0.807(0.592-1.099)	0.174	0.948(0.630-1.426)	0.797	0.945(0.379-2.354)	0.903
**Sex**								
Male vs Female	0.993(0.807-1.221)	0.944	0.967(0.746-1.254)	0.802	1.153(0.812-1.639)	0.426	1.301(0.596-2.840)	0.508
**Grade**								
II vs I	1.271(0.795-2.034)	0.317	2.216(0.989-4.967)	0.053	2.781(0.650-11.894)	0.168	0.697(0.073-6.638)	0.754
III vs I	1.623(1.024-2.572)	0.039	3.568(1.615-7.885)	0.002	6.141(1.473-25.363)	0.013	1.742(0.207-14.648)	0.610
IV vs I	1.006(0.437-2.318)	0.988	1.275(0.347-4.690)	0.715	3.894(0.666-22.780)	0.131	4.672(0.394-55.351)	0.222
unknown vs I	0.967(0.444-2.109)	0.934	2.904(1.039-8.116)	0.042	2.850(0.629-1.363)	0.267	2.954(0.210-41.530)	0.422
**Type of gastrectomy**								
Subtotal vs Total	1.270(0.962-1.677)	0.091	0.979(0.715-1.340)	0.893	1.324(0.834-2.102)	0.234	0.871(0.360-2.108)	0.760
**Year of Diagnosis**								
2006-07 vs 2004-05	0.914(0.659-1.266)	0.587	0.982(0.658-1.467)	0.931	0.931(0.525-1.649)	0.806	1.822(0.358-9.278)	0.470
2008-09 vs 2004-05	0.952(0.685-1.323)	0.770	0.916(0.605-1.386)	0.678	1.195(0.684-2.088)	0.530	0.539(0.069-4.198)	0.555
2010-11 vs 2004-05	0.920(0.682-1.243)	0.588	0.883(0.606-1.287)	0.519	0.837(0.488-1.436)	0.517	2.308(0.491-10.841)	0.289
2012-14 vs 2004-05	0.929(0.679-1.271)	0.644	0.916(0.613-1.368)	0.668	0.931(0.516-1.678)	0.811	3.473(0.691-17.463)	0.131

Table S11. Multivariate logistic analysis of patient and tumor characteristics in T3 stage of SEER dataset.

	N1 vs N0		N2 vs N0		N3a vs N0		N3b vs N0	
	OR(95%)	*P*	OR(95%)	*P*	OR(95%)	*P*	OR(95%)	*P*
**Number of LN examined (continuous)**								
Log transformed	0.929(0.758-1.139)	0.479	2.738(2.189-3.425)	0.000	11.712(8.880-15.449)	0.000	908(511-1614)	0.000
**Age**								
18-39 yrs	1.294(0.778-2.152)	0.321	1.255(0.762-2.066)	0.372	1.821(1.122-2.956)	0.015	1.701(0.841-3.440)	0.139
40-49 yrs	0.980(0.734-1.308)	0.889	0.792(0.590-1.062)	0.119	1.074(0.801-1.438)	0.634	1.048(0.667-1.647)	0.840
50-59 yrs	0.978(0.777-1.231)	0.849	1.057(0.847-1.319)	0.626	1.051(0.833-1.327)	0.676	0.937(0.651-1.349)	0.725
**60-69 yrs**	**Reference**		**Reference**		**Reference**		**Reference**	
70-79 yrs	0.889(0.736-1.074)	0.224	0.837(0.693-1.009)	0.063	0.801(0.654-0.982)	0.033	0.927(0.667-1.289)	0.652
80+ yrs	0.810(0.659-0.997)	0.047	0.769(0.625-0.947)	0.013	0.666(0.528-0.840)	0.001	0.806(0.545-1.193)	0.281
**Race**								
Black vs White	1.164(0.955-1.418)	0.134	1.134(0.932-1.381)	0.210	1.001(0.804-1.246)	0.993	1.117(0.788-1.584)	0.534
Other vs White	1.116(0.930-1.338)	0.239	0.900(0.748-1.083)	0.265	1.063(0.878-1.286)	0.531	1.114(0.830-1.496)	0.472
**Sex**								
Male vs Female	0.889(0.769-1.027)	0.110	0.875(0.757-1.011)	0.069	0.878(0.752-1.026)	0.101	0.983(0.766-1.261)	0.890
**Grade**								
II vs I	1.183(0.799-1.753)	0.401	2.144(1.318-3.486)	0.002	1.424(0.838-2.420)	0.191	2.748(0.616-12.256)	0.185
III vs I	1.439(0.982-2.109)	0.062	3.066(1.904-4.938)	0.000	3.181(1.902-5.318)	0.000	11.060(2.551-47.954)	0.001
IV vs I	1.321(0.756-2.306)	0.328	2.875(1.551-5.330)	0.001	2.896(1.499-5.593)	0.002	7.797(1.504-40.416)	0.014
unknown vs I	1.453(0.848-2.488)	0.174	2.111(1.121-3.975)	0.021	2.101(1.062-4.154)	0.033	12.454(2.519-61.58)	0.002
**Type of gastrectomy**								
Subtotal vs Total	0.925(0.780-1.098)	0.374	0.985(0.832-1.165)	0.856	0.899(0.755-1.070)	0.230	0.641(0.497-0.826)	0.001
**Year of Diagnosis**								
2006-07 vs 2004-05	0.995(0.790-1.253)	0.964	0.824(0.657-1.033)	0.093	0.856(0.673-1.088)	0.204	0.717(0.490-1.048)	0.085
2008-09 vs 2004-05	0.982(0.784-1.230)	0.874	0.749(0.600-0.935)	0.011	0.676(0.533-0.859)	0.001	0.548(0.376-0.798)	0.002
2010-11 vs 2004-05	0.842(0.679-1.045)	0.119	0.662(0.536-0.817)	0.000	0.560(0.445-0.703)	0.000	0.378(0.263-0.545)	0.000
2012-14 vs 2004-05	1.253(1.001-1.568)	0.049	1.141(0.916-1.422)	0.240	1.161(0.916-1.472)	0.217	0.926(0.629-1.363)	0.695

Table S12. Multivariate logistic analysis of patient and tumor characteristics in T4 stage of SEER dataset.

	N1 vs N0		N2 vs N0		N3a vs N0		N3b vs N0	
	OR (95%)	*P*	OR (95%)	*P*	OR (95%)	*P*	OR (95%)	*P*
**Number of LN examined (continuous)**								
Log transformed	0.895(0.689-1.162)	0.406	2.986(2.274-3.921)	0.000	20.490(14.828-28.314)	0.000	1876(1083-3249)	0.000
**Age**								
18-39 yrs	1.361(0.778-2.381)	0.281	1.073(0.640-1.800)	0.788	0.988(0.587-1.661)	0.962	0.530(0.269-1.043)	0.066
40-49 yrs	1.177(0.799-1.733)	0.410	0.935(0.654-1.337	0.712	0.908(0.632-1.305)	0.603	0.839(0.538-1.308)	0.439
50-59 yrs	0.993(0.727-1.355)	0.962	0.797(0.599-1.061)	0.121	0.999(0.752-1.328)	0.997	0.764(0.534-1.094)	0.142
**60-69 yrs**	**Reference**		Reference		Reference		Reference	
70-79 yrs	1.075(0.821-1.407)	0.599	0.782(0.609-1.004)	0.054	1.017(0.790-1.309)	0.899	0.996(0.726-1.367)	0.982
80+ yrs	1.287(0.961-1.722)	0.090	0.961(0.729-1.266)	0.777	1.106(0.832-1.470)	0.487	1.164(0.810-1.674)	0.411
**Race**								
Black vs White	1.422(1.075-1.881)	0.014	1.404(1.070-1.842)	0.014	1.417(1.074-1.869)	0.014	1.150(0.800-1.652)	0.451
Other vs White	0.909(0.707-1.168)	0.454	1.133(0.902-1.423)	0.282	1.170(0.933-1.476)	0.175	1.056(0.797-1.400)	0.705
**Sex**								
Male vs Female	0.945(0.779-1.148)	0.570	0.879(0.731-1.056)	0.168	0.832(0.692-1.001)	0.051	0.852(0.675-1.076)	0.180
**Grade**								
II vs I	1.232(0.694-2.186)	0.477	1.719(0.917-3.220)	0.091	3.535(1.407-8.879)	0.007	2.729(0.587-12.678)	0.200
III vs I	1.672(0.965-2.897)	0.067	2.951(1.609-5.412)	0.000	9.599(3.893-23.669)	0.000	12.346(2.736-55.697)	0.001
IV vs I	0.747(0.327-1.704)	0.488	2.198(0.999-4.837)	0.050	8.150(2.913-22.805)	0.000	21.946(4.380-109.97)	0.000
unknown vs I	1.441(0.712-2.919)	0.310	1.772(0.827-3.798)	0.141	7.395(2.709-20.189)	0.000	8.770(1.723-44.646)	0.009
**Type of gastrectomy**								
Subtotal vs Total	1.173(0.934-1.473)	0.169	1.262(1.020-1.562)	0.032	0.940(0.766-1.153)	0.552	0.737(0.576-0.944)	0.016
**Year of Diagnosis**								
2006-07 vs 2004-05	0.762(0.570-1.018)	0.066	0.770(0.582-1.018)	0.067	0.701(0.530-0.929)	0.013	0.950(0.659-1.369)	0.782
2008-09 vs 2004-05	0.893(0.649-1.229)	0.488	0.916(0.675-1.244)	0.575	0.800(0.589-1.087)	0.154	0.954(0.645-1.412)	0.816
2010-11 vs 2004-05	0.828(0.619-1.106)	0.201	0.892(0.676-1.176)	0.417	0.738(0.559-0.973)	0.031	0.734(0.513-1.050)	0.091
2012-14 vs 2004-05	0.880(0.660-1.175)	0.386	1.144(0.869-1.506)	0.338	1.123(0.850-1.483)	0.415	1.395(0.967-2.011)	0.075

Table S13. Rate ratio estimates from multivariable Poisson model for number of positive LN in China dataset.

	Final Stage = T1		Final Stage = T2		Final Stage = T3		Final Stage = T4	
	**Rate Ratio (95%)**	***P***	**Rate Ratio (95%)**	***P***	**Rate Ratio (95%)**	***P***	**Rate Ratio (95%)**	***P***
**Age**								
**Ref group is ages 60-69**								
18-39 yrs	2.979 (2.484-3.572)	<0.0001	1.850 (1.555-2.200)	<0.0001	1.662 (1.494-1.849)	<0.0001	1.341 (1.278-1.407)	<0.0001
40-49 yrs	1.317 (1.084-1.602)	0.006	0.556 (0.448-0.693)	<0.0001	0.973 (0.860-1.102)	0.667	1.132 (1.085-1.180)	<0.0001
50-59 yrs	0.818 (0.674-0.993)	0.042	0.977 (0.841-1.134)	0.758	1.058 (0.964-1.134)	0.233	1.143 (1.104-1.184)	<0.0001
**60-69 yrs**	**Reference**		**Reference**		**Reference**		**Reference**	
70+ yrs	0.958 (0.746-1.230)	0.737	0.639 (0.512-0.798)	<0.0001	1.015 (0.898-1.147)	0.809	1.100 (1.050-1.152)	<0.0001
**Number of LN examined (continuous)**								
Log transformed	5.056 (3.953-6.466)	<0.0001	3.028 (2.367-3.874)	<0.001	5.483 (4.655-6.459)	<0.0001	7.582 (7.155-8.034)	<0.0001
**Year of Diagnosis**								
2006-07 vs 2004-05	0.453 (0.345-0.594)	0.041	0.606 (0.451-0.812)	0.0008	6.115 (3.741-9.994)	<0.0001	0.940 (0.886-0.997)	0.041
2008-09 vs 2004-05	0.237 (0.184-0.304)	<0.0001	0.670 (0.520-0.863)	0.0019	3.971 (2.519-6.257)	<0.0001	0.829 (0.788-0.871)	<0.0001
2010-11 vs 2004-05	0.384 (0.314-0.470)	<0.0001	0.648 (0.504-0.833)	0.0007	5.600 (3.731-8.405)	<0.0001	0.901 (0.857-0.948)	<0.0001
2012-14 vs 2004-05	0.180 (0.151-0.214)	<0.0001	0.434 (0.334-0.564)	<0.001	3.584 (2.385-5.388)	<0.0001	0.629 (0.599-0.661)	<0.0001
**Sex (Male vs Female)**	1.043 (0.910-1.196)	0.546	0.690 (0.611-0.779)	<0.0001	0.965 (0.894-1.042)	0.361	0.954 (0.926-0.982)	0.0013
**Grade**								
II vs I	0.731 (0.561-0.953)	0.02	0.668 (0.516-0.866)	0.002	0.446 (0.375-0.529)	<0.0001	1.033 (0.949-1.123)	0.453
III/IV/unknown vs I	1.246 (1.000-1.552)	0.05	0.894 (0.706-1.132)	0.353	0.750 (0.641-0.876)	0.0003	1.338 (1.235-1.449)	<0.0001
**Type of gastrectomy**								
Subtotal vs Total	0.592 (0.513-0.682)	<0.0001	1.332 (1.146-1.547)	0.0002	0.944 (0.876-1.017)	0.129	0.830 (0.808-0.853)	<0.0001

Table S14. Rate ratio estimates from a single multivariable Poisson model for number of positive LN for stage T1 in SEER dataset.

	Final Stage = T1	
	**Rate Ratio (95%)**	***P***
**Age - Race**		
**Ref group is White, ages 60-69**		
**White**		
18-39 yrs	1.341 (1.010-1.782)	0.043
40-49 yrs	1.050 (0.845-1.304)	0.661
50-59 yrs	0.895 (0.759-1.054)	0.184
**60-69 yrs**	**Reference**	
70-79 yrs	1.032 (0.913-1.168)	0.612
80+ yrs	1.515 (1.318-1.743)	<0.001
**Black**		
20-39 yrs	0.582 (0.185-1.829)	0.354
40-49 yrs	2.029 (1.496-2.753)	<0.001
50-59 yrs	0.646 (0.484-0.861)	0.003
60-69 yrs	1.144 (0.910-1.439)	0.248
70-79 yrs	1.022 (0.806-1.296)	0.859
80+ yrs		
**Other**		
20-39 yrs	0.971 (0.569-1.655)	0.912
40-49 yrs	1.644 (1.303-2.076)	<0.001
50-59 yrs	1.185 (0.968-1.452)	0.101
60-69 yrs	1.029 (0.854-1.239)	0.763
70-79 yrs	1.025 (0.869-1.209)	0.768
80+ yrs	0.929 (0.739-1.168)	0.528
**Number of LN examined (continuous)**		
Log transformed	4.091 (3.572-4.686)	<0.0001
**Year of Diagnosis**		
2006-07 vs 2004-05	0.845 (0.744-0.960)	0.010
2008-09 vs 2004-05	0.567 (0.499-0.651)	<0.0001
2010-11 vs 2004-05	0.642 (0.570-0.724)	<0.0001
2012-14 vs 2004-05	0.984 (0.869-1.116)	0.807
**Sex (Male vs Female)**	0.899 (0.831-0.973)	0.008
**Grade**		
II vs I	1.053 (0.843-1.316)	0.649
III/IV/unknown vs I	1.235 (0.995-1.533)	0.056
**Type of gastrectomy**		
Subtotal vs Total	1.236 (1.109-1.377)	0.0001

Table S15. Rate ratio estimates from multivariable Poisson model for number of positive LN in SEER dataset.

	**Final Stage = T2**		**Final Stage=T3**		**Final Stage=T4**	
	**Rate Ratio (95%)**	***P***	**Rate Ratio (95%)**	***P***	**Rate Ratio (95%)**	***P***
**Age**						
**Ref group is ages 60-69**						
18-39 yrs	1.771 (1.073-2.923)	0.025	1.111 (1.042-1.185)	0.0013	1.239 (1.161-1.323)	<0.0001
40-49 yrs	1.250 (0.920-1.698)	0.153	0.972 (0.931-1.015)	0.202	1.225 (1.152-1.302)	<0.0001
50-59 yrs	1.096 (0.872-1.377)	0.434	0.989 (0.956-1.022)	0.504	1.276 (1.202-1.354)	<0.0001
**60-69 yrs**	**Reference**		**Reference**		**Reference**	
70-79 yrs	0.645 (0.517-0.806)	0.0001	0.949 (0.920-0.979)	0.001	1.296 (1.222-1.376)	<0.0001
80+ yrs	0.976 (0.780-1.221)	0.83	0.903 (0.871-0.937)	<0.0001	1.293 (1.215-1.375)	<0.0001
**Number of LN examined (continuous)**						
Log transformed	1.225 (0.990-1.514)	0.061	6.338 (6.079-6.608)	<0.0001	7.509 (7.225-7.805)	<0.0001
**Race**						
Black vs White	1.813 (1.218-2.696)	0.003	1.008 (0.975-1.041)	0.652	0.990 (0.959-1.022)	0.550
Other vs White	1.791 (1.397-2.295)	<0.0001	1.042 (1.013-1.072)	0.004	1.028 (1.003-1.054)	0.028
**Year of Diagnosis**						
2006-07 vs 2004-05	1.509 (1.199-1.898)	0.0004	0.889 (0.857-0.921)	<0.0001	1.006 (0.973-1.040)	0.725
2008-09 vs 2004-05	0.890 (0.719-1.102)	0.286	0.827 (0.798-0.857)	<0.0001	0.969 (0.936-1.003)	0.076
2010-11 vs 2004-05	1.112 (0.890-1.375)	0.325	0.746 (0.721-0.772)	<0.0001	0.913 (0.885-0.943)	<0.0001
2012-14 vs 2004-05	0.970 (0.784-1.199)	0.779	0.983 (0.950-1.018)	0.348	1.097 (1.062-1.132)	<0.0001
**Sex (Male vs Female)**	2.500 (1.825-3.425)	<0.0001	1.007 (0.983-1.031)	0.581	1.022 (1.001-1.044)	0.040
**Grade**						
II vs I	1.184 (0.823-1.703)	0.362	1.322 (1.192-1.466)	<0.0001	1.603 (1.389-1.850)	<0.0001
III/IV/unk vs I	1.127 (0.876-1.450)	0.351	1.992 (1.801-2.203)	<0.0001	2.311 (2.009-2.659)	<0.0001
**Type of gastrectomy**						
Subtotal vs Total	0.956 (0.771-1.186)	0.681	0.885 (0.864-0.908)	<0.0001	0.859 (0.841-0.878)	<0.0001
